# Tunable Multilevel Data Storage Bioresistive Random Access Memory Device Based on Egg Albumen and Carbon Nanotubes

**DOI:** 10.3390/nano11082085

**Published:** 2021-08-17

**Authors:** Lu Wang, Tianyu Yang, Dianzhong Wen

**Affiliations:** HLJ Province Key Laboratory of Senior-Education for Electronic Engineering, School of Electronic Engineering, Heilongjiang University, Harbin 150080, China; 2201633@s.hlju.edu.cn (T.Y.); wendianzhong@hlju.edu.cn (D.W.)

**Keywords:** egg albumen, carbon nanotubes, multilevel data storage, tuneable resistive random access memories

## Abstract

In this paper, a tuneable multilevel data storage bioresistive memory device is prepared from a composite of multiwalled carbon nanotubes (MWCNTs) and egg albumen (EA). By changing the concentration of MWCNTs incorporated into the egg albumen film, the switching current ratio of aluminium/egg albumen:multiwalled carbon nanotubes/indium tin oxide (Al/EA:MWCNT/ITO) for resistive random access memory increases as the concentration of MWCNTs decreases. The device can achieve continuous bipolar switching that is repeated 100 times per cell with stable resistance for 10^4^ s and a clear storage window under 2.5 × 10^4^ continuous pulses. Changing the current limit of the device to obtain low-state resistance values of different states achieves multivalue storage. The mechanism of conduction can be explained by the oxygen vacancies and the smaller number of iron atoms that are working together to form and fracture conductive filaments. The device is nonvolatile and stable for use in rewritable memory due to the adjustable switch ratio, adjustable voltage, and nanometre size, and it can be integrated into circuits with different power consumption requirements. Therefore, it has broad application prospects in the fields of data storage and neural networks.

## 1. Introduction

Because von Neumann theory makes the development of modern technology difficult, resistive random-access memories (RRAMs) with storage and computing properties have been candidate devices to solve this problem. RRAMs have a simple structure, low power consumption, high integration potential, and fast read and write speeds; they also have multivalue computing potential and are very compatible with complementary metal oxide semiconductor (CMOS) technology [1]. At present, the research status of RRAM has been extended to many application technology fields because it can exhibit synaptic behaviour such as short-term plasticity (STP), long-term plasticity (LTP), spike-time-dependent plasticity (STDP), paired-pulse facilitation (PPF), and paired-pulse depression (PPD) [2]. This technology can be applied to neural networks and artificial intelligence; the RRAM of the crossbar array can store and calculate integrated memory; it can be used in chaotic circuits to form a confidentiality system [3]. RRAM made for biological applications can be applied to wearable devices [4]. The characteristics and advantages of RRAMs are closely related to the materials used for making the devices, so the choice of materials is vital. RRAMs made of inorganic materials are fragile, incompatible, and difficult to degrade, which leads to immense challenges [5]. Great progress has been made in the fabrication of functional electronic devices using organic biomaterials, and a new generation of environmentally friendly and biocompatible natural bioorganic materials as functional layers of RRAM has attracted increasing attention. Natural bioorganic materials such as nanocellulose [6], DNA [7,8], protein, chitosan [9], and starch [10] have been used in the fabrication of RRAM.

The biodegradability and bioabsorbability of protein-based biological RRAMs make them applicable for bioimplantable medical devices. They are often used in flexible wearable electronic devices due to their light weight, stretchability, and foldability. Protein is nonhazardous, nonpolluting, easy to obtain, and can be mass produced at low cost. Protein-based RRAMs of silk fibroin [11], silkworm haemolymph [12], sericin [13], and egg protein [14,15] have been proposed. As a natural biological material, egg white does not require additional chemical extraction and purification processes, which reduces manufacturing costs and simplifies processing steps. In 2015, Chen fabricated Al/EA/ITO RRAMs using egg white as the active layer and found that the devices have nonvolatile bipolar switching characteristics [16]. Qu proposed a novel one-write-multiread RRAM based on a protein film, forming an Ag/EA/ITO/polyethylene terephthalate (PET) structure [17]. Wang controlled the current switching ratio of Al/graphene oxide (GO)/EA/GO/ITO devices using varying concentrations of GO [18]. Other studies have demonstrated that RRAMs made from egg proteins are water soluble [19], can successfully simulate the memory behaviour of biological synapses [20,21,22], can be flexibly transferred to many unconventional substrates [23], and can realize the full memory logic block of the non-gate, or-gate, and and-gate [24]. Egg proteins can also be made into gate dielectric layers of organic field effect transistors and other transistors [25,26]. Therefore, egg protein RRAMs are competitive as biological RRAMs and have very broad research prospects.

The egg protein RRAMs in the literature have a switching ratio of 10^2^ to 10^7^, and their resistance switching characteristics vary greatly. Therefore, it is valuable to find a way to reliably adjust the resistance switching characteristics of the device. The bipolar resistance switching behaviour is the result of the formation and rupture of conductive filaments caused by ion migration in the active layer of the egg protein RRAM. The concentration of migrating ions in the active layer could be precisely controlled to affect the resistance switching behaviour of the biological protein RRAM. The concentration of doping in the active layer greatly affects the concentration of migrating ions. However, there is a lack of relevant research on adjustment of the doping level to significantly change the resistance switching characteristics of egg protein RRAMs.

As nanoscale structures of carbon, carbon nanotubes have excellent electrical, mechanical, thermal, and hydrogen storage properties; [27] therefore, they are used in new electronic sources [28], sensors [29], composite materials [30], stealth materials [31], and hydrogen storage materials [32]. The orientation, arrangement, and additional conditions of the MWCNTs added to the source layer result in RRAMs with novel characteristics [33,34]. Liu used a conjugated polymer mixed with carbon nanotubes to form the active layer of RRAM, which exhibits a switching behaviour of one write and many reads [35]. Kim developed a carbon nanotube RRAM with basic dynamic logic, learning and memory functions for biological synapses [36]. Molinari used a nanocomposite of polycaprolactone, multiwalled carbon nanotubes, and fullerene C_60_ as a memristor layer to create a one-write-multiread memory device, and the resistance of the device can be modulated by applying different programming voltages and programming times [37].

At present, there are few studies about combining natural biomaterials with carbon nanomaterials to make an active layer for RRAMs. To improve the performance of RRAM, nanoparticles were added into the natural organic active layer to overcome the limitations of the single active layer. In this paper, multiwalled carbon nanotubes (MWCNTs) were added to egg white to form nanocomposites, and active thin films were fabricated by a simple spin-coating process to obtain aluminium/egg albumen: multiwalled carbon nanotube/indium tin oxide (Al/EA: MWCNT/ITO) devices. The switching current ratio and reset voltage could be adjusted via the concentration of MWCNTs. The addition of MWCNTs to the active layer made the high and low resistances of the device more uniform.

## 2. Materials and Methods

### 2.1. Fabrication of the Flexible Memristor

Chicken eggs were purchased in local supermarkets. Carboxylated MWCNTs, purity >95 ωt%, carboxyl content 2.00 ωt%, inner diameter 5–10 nm, outer diameter 10–20 nm, and length 10–30 µm, were purchased from Suzhou Tanfeng Graphene Technology Company (Suzhou, China). The egg albumen obtained from eggs was mixed with deionized water at a ratio of 1:15 and ultrasonicated for 15 min. A mixture of 1.2 ωt%, 3 ωt%, and 6 ωt% MWCNTs and deionized water was treated by ultrasonication for more than 5 h to uniformly disperse the MWCNTs in the liquid. The egg albumen solution and MWCNT dispersion were prepared at a ratio of 1:1 by ultrasonication for 15 min until the mixture was completely mixed. The mixed solution was spin-coated on glass coated with indium tin oxide film, which was washed by ultrasonication for 15 min in alcohol, acetone, and deionized water; the spin-coating method comprised an initial rotation speed of 500 rpm, a rotation time of 5 s, a final rotation speed of 4000 rpm, and a rotation time of 60 s. The device was dried at 105 °C for 15 min, and the upper aluminium electrode was vapour-deposited on the active layer. Finally, the device was annealed at 105 °C for 15 min to complete the fabrication of the Al/EA:MWCNT/ITO device.

### 2.2. Characterizations and Electrical Measurements of the Memristor

Using a scanning electron microscope (SEM, Hitachi S3400) (Hitachi, Tokyo, Japan), the cross section of EA: MWCNTs/ITO was observed. Energy dispersive spectroscopy analysis of the chemical elements in the EA/ITO/glass and EA: MWCNTs/ITO/glass was performed. An ultraviolet-visible (UV-Vis) spectrophotometer (UV/VIS, TU-1901) (Puxi, Beijing, China) was used to measure the UV-Vis spectra of the egg film and MWCNT film. A transmission electron microscope (TEM, JEM-2100) (JEOL, Tokyo, Japan) was used to observe the microstructure of the MWCNTs. A semiconductor parameter tester (Keithley 4200) (Keithley, Solon, OH, USA) was used to test the I-V characteristics of the Al/EA:MWCNT/ITO device.

## 3. Results and Discussion

### 3.1. Device Structure and Material Characterization

**Figure 1:**
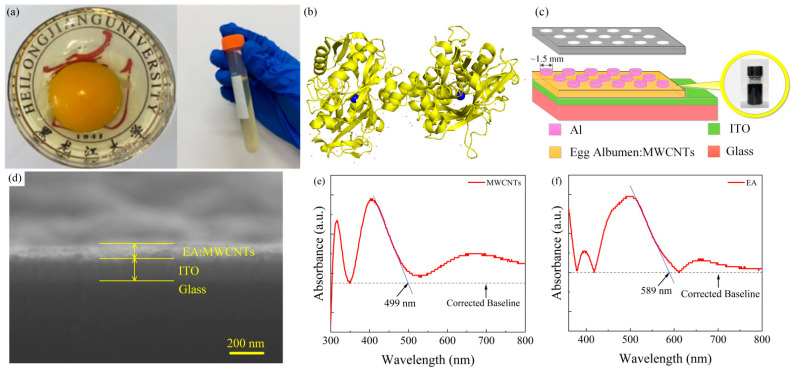
(**a**) The egg white and yolk of an egg and the egg white taken from it. (**b**) Three−dimensional ribbon model of eggs with double egg transferrin. (**c**) Schematic configuration of the Al/EA: MWCNT/ITO device. (**d**) Cross−section of the device. UV−Vis absorption spectra of the (**e**) MWCNT film and (**f**) egg albumen film.

Figure 1a shows the egg white and yolk obtained from fresh eggs, and the albumen liquid was collected in a centrifuge tube without any subsequent processing. Ovotransferrin accounts for approximately 12–13% of the total egg protein [38], and Figure 1b shows the three-dimensional ribbon model of the egg’s double ovotransferrin. It is a polymer composed of 5284 protein atoms (yellow ribbons and yellow thread), tw trivalent iron ions (two blue balls), and 132 water molecules (red dots) [39]. Figure 1c shows the structural model of the Al/EA: MWCNT/ITO device and composite material based on MWCNTs and egg albumen collected from glass vials. The diameter of each upper electrode is approximately 1.5 mm. Figure 1d shows a cross-section of the device under a scanning electron microscope. The thickness of the active layer of the composite material of MWCNTs and egg albumen is approximately 120 nm, and the thickness of the ITO layer is approximately 200 nm. The thickness of the Al electrode was approximately 200 nm, and the diameter of each cell was approximately 1.5 mm. As shown in Figure 1e,f, the MWCNT film and egg film were optically analysed by UV-visible spectroscopy. The wavelength corresponding to Eg is obtained by the intersection of the edge tangent of the absorption peak and the corrected baseline, so the absorption peak edges of the MWCNT film and the egg film are at λ = 499 nm and λ = 589 nm, respectively. According to the equation Eg = hc/λ, the band gaps of MWCNTs and eggs are approximately 2.48 eV and 2.11 eV, respectively. For the calculation, Planck’s constant h = 6.62 × 10^−34^ Js and the propagation speed of light in a vacuum c = 3 × 10^8^ m/s were used [40].

**Figure 2:**
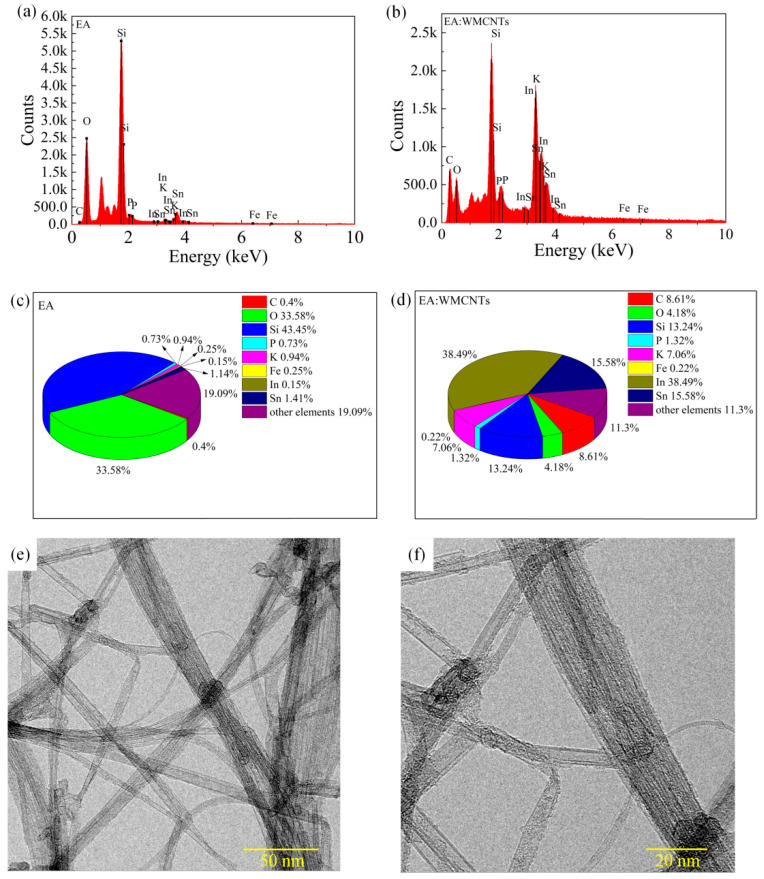
EDS spectra of (**a**) EA/ITO/glass and (**b**) EA: 1.2 ωt% MWCNTs/ITO/glass. Element content statistical analysis of (**c**) EA/ITO/glass and (**d**) EA: 1.2 ωt% MWCNTs/ITO/glass. TEM images of MWCNTs: (**e**) low resolution and (**f**) high resolution.

Figure 2a,b shows the EDS spectra of the EA/ITO/glass and EA: 1.2 ωt% MWCNTs/ITO/glass doped with carbon nanotubes. The peak value of C in the EA:1.2 ωt% film is significantly higher than that in the EA film. In Figure 2c,d, the content of C in the film increased from 0.4% to 8.61%, which proves that the carbon nanotubes were doped into the egg white film. We also performed TEM mapping characterization tests on carbon nanotubes. Figure 2e,f show the microstructures of carbon nanotubes at different resolutions.

### 3.2. Memristor Performance

**Figure 3:**
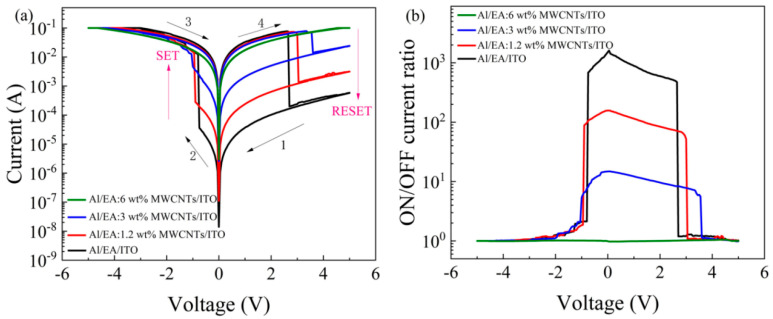
(**a**) The I−V characteristics and (**b**) switching current ratio of Al/EA/ITO and Al/EA: (1.2 ωt%, 3 ωt%, and 6 ωt%) MWCNTs/ITO.

To study the influence of different concentrations of MWCNTs on the electrical characteristics of Al/EA: MWCNT/ITO devices, the I–V scanning characteristic curve of the devices was analysed. The scan voltage range was −5.00–5.00 V, and the limit current Icc was 0.1 A. Figure 3a shows that Al/EA/ITO and Al/EA: (1.2 ωt%, 3 ωt%, 6 ωt%) MWCNT/ITO devices have bipolar resistance switching behaviour, and the switching current ratio increases as the concentration of MWCNTs doped into the active layer decreases. Figure 3b shows the adjustability of the device switching current ratio. At a voltage of 0.1 V, the switching current ratios of the Al/EA/ITO and Al/EA: (1.2 ωt%, 3 ωt%, and 6 ωt%) MWCNT/ITO devices were 1.42 × 10^3^, 1.56 × 10^2^, 14, and 1, respectively.

**Figure 4:**
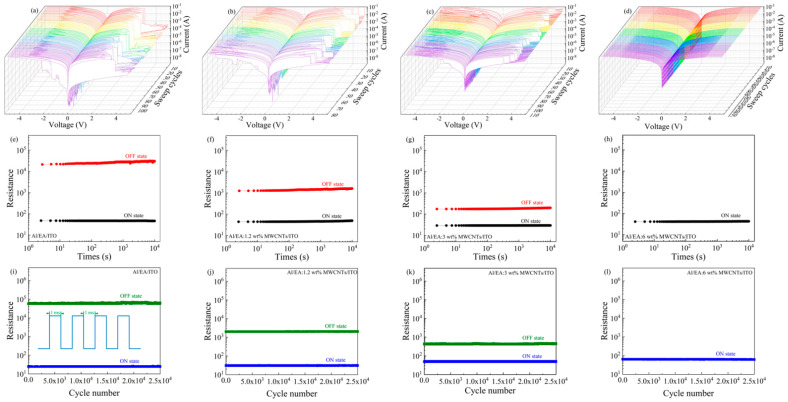
(**a**–**d**) The I−V characteristics under continuous cycle scanning and (**e**–**h**) the retention times and (**i**–**l**) endurance cycles of Al/EA/ITO and Al/EA: (1.2 ωt%, 3 ωt%, 6 ωt%) MWCNTs/ITO.

As shown in Figure 4a–d, I-V scanning of the same cell could be repeated more than 100 times for the Al/EA/ITO and Al/EA: (3 ωt%, 6 ωt%) MWCNT/ITO devices, and scanning of the Al/EA:1.2 ωt% MWCNT/ITO device could be repeated 70–80 times. Their switch ratio window size remained basically unchanged, and the high- and low-resistance states were relatively stable. Figure 4e–h shows the measurement of the retention time of the four devices. Under a 1 V DC bias, the high and low resistance state.

Values after writing or erasing were relatively stable, and the holding time reached 10^4^ s, which reflects the strong holding ability of the device. As shown in Figure 4i–l, four devices were tested for voltage pulses. The pulse period was 2 ms, the duty cycle was 50%, and the amplitude was 1 V. After 2.5 × 10^4^ continuous pulses, the device still had a clear write/erase window, which reflects the good durability of the device.

**Figure 5:**
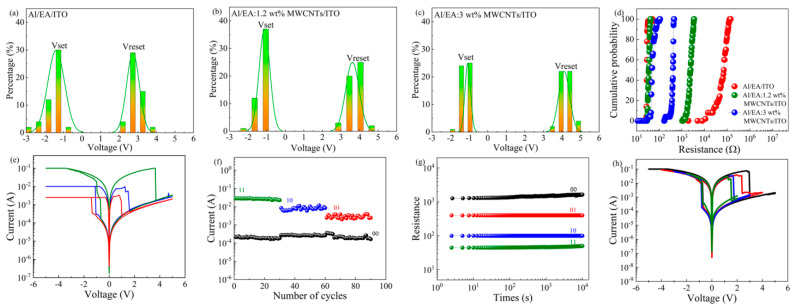
(**a**–**c**) Statistical distribution of Vset and Vreset and (**d**) the cumulative probability of the resistance of Al/EA/ITO and Al/EA: (1.2 ωt%, 3 ωt%) MWCNTs/ITO. (**e**–**h**) Al/EA: 1.2 ωt% MWCNTs/ITO: (**e**) I−V curve under different compliance currents, (**f**) Continuous cycle I−V characteristics under different compliance currents, (**g**) Four state retention characteristics, (**h**) I−V curve under different scanning voltage ranges.

As shown in Figure 5a–c, the write/erase process and the stability of the high and low resistance states were tested and analysed. The SET voltage (Vset) and RESET voltage (Vreset) of the first 50 I-V cycles of the Al/EA/ITO and Al/EA: (1.2 ωt%, 3 ωt%) MWCNT/ITO devices were statistically distributed on a histogram. The cumulative probability of high resistance state resistance (R_HRS_) and low resistance state resistance (R_LRS_) are shown in Figure 5d. The median Vset of the three devices was −1.33 V, −1.08 V, and −1.08 V, respectively, and the median Vreset was 2.70 V, 3.68 V and 4.13 V, respectively. With the increase in the concentration of MWCNTs incorporated in the egg albumen membrane, Vreset increased continuously, but the effect on Vset was small.

The median R_HRS_ of the three devices was 7.18 × 10^4^ Ω, 2.43 × 10^3^ Ω and 4.05 × 10^2^ Ω, and the coefficients of variation were 0.45, 0.22 and 0.14, respectively. The median R_LRS_ was 28.31 Ω, 34.80 Ω, and 49.44 Ω, and the coefficients of variation were 0.11, 0.09, and 0.24, respectively. As the concentration of MWCNTs increased, the degree of dispersion of the R_HRS_ probability distribution decreased, and the R_LRS_ of the Al/EA: 1.2 ωt% MWCNT/ITO device was more concentrated than that of the other two devices. Therefore, the egg albumen RRAM with incorporated MWCNTs is more reliable.

In this paper, the coordinated Al/EA:MWCNT/ITO device could achieve multilevel data storage to improve the storage density. Different limiting currents can be set on the same unit of the device to obtain different switching current ratios, which realizes 2-bit memory. As shown in Figure 5e, under a negative scanning voltage, the Al/EA: 1.2 ωt% MWCNT/ITO device was set to different limit currents of 100 mA, 10 mA and 2.5 mA, and the limit current of the positive scanning voltage was uniformly set to 100 mA. The different switch ratios were 122, 30, and 9. The R_LRS_ was largely affected by the limited current, whereas the R_HRS_ was basically unchanged. Figure 5f shows the stability and reliability of the device, which achieved multilevel data storage. The same cell of the device performed 30 consecutive read and write cycles at a voltage of 1 V. Figure 5g shows the retention capability of the multilevel data storage of the test device, and the data were stable for 10^4^ s under 1 V. The multilevel data storage mechanism indicates that the device would produce fewer conductive filaments under a larger limiting current. When the number of conductive filaments was reduced, a smaller Vreset caused the device to change from a low resistance state to a high resistance state. Therefore, the Vreset voltage decreased with increasing the compliance currents, and the switching ratio decreased accordingly. As shown in Figure 5h, the device maintained read and write capabilities under different scan voltage ranges to achieve low-power storage.

### 3.3. Mechanism of the Memristive Switch

**Figure 6:**
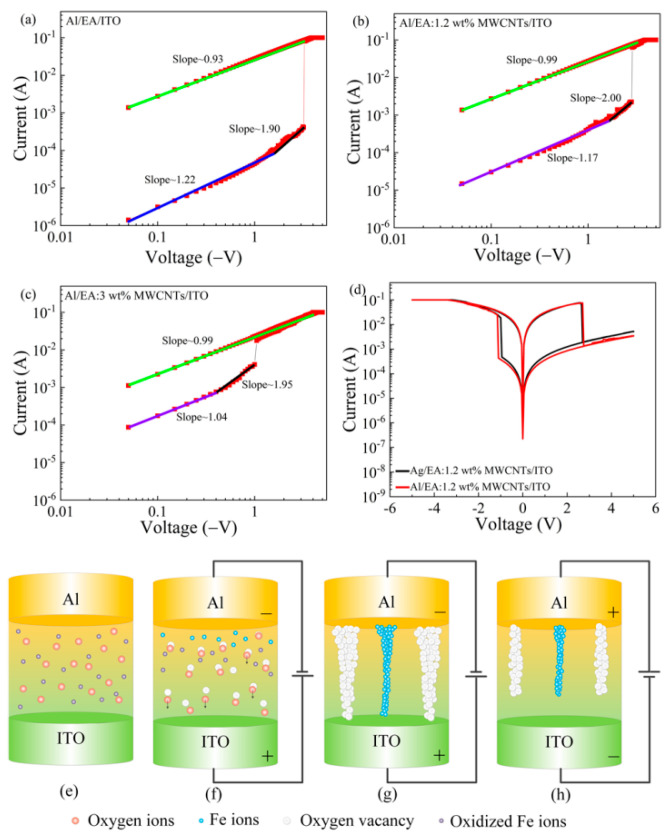
(**a**–**c**) Al/EA/ITO and Al/EA: (1.2 ωt%, 3 ωt%) MWCNT/ITO double logarithmic fitting curves of I-V under negative bias. (**d**) I-V characteristics of Ag/EA: 1.2 ωt% MWCNTs/ITO and Al/EA: 1.2 ωt% MWCNTs/ITO. (**e**–**h**) Resistance switching mechanism model.

To explore the current transmission mechanism of the device, the I-V scan curve of the device was redrawn with double logarithmic coordinates under negative voltage, as shown in Figure 6a–c. In a low resistance state (LRS), the slopes of the corresponding double logarithmic (I–V) curves of the device, Al/EA/ITO and Al/EA: (1.2 ωt%, 3 ωt%) MWCNTs/ITO, are 0.93, 0.99, and 0.99, respectively, which are all close to 1, and the conduction behaviour of carriers in this region obeys Ohm’s law [41]. In the low voltage range of the high resistance state (HRS), the corresponding slopes are 1.22, 1.17, and 1.04, and this region is represented as an ohmic conductive region. Under the HRS and high electric field with increasing voltage, the slopes are 1.90, 2.00, and 1.95, which are all close to 2. This region is expressed as the Child’s law region, and the current conforms to the Mott–Gurney law. The current transmission of the device conforms to the space charge limited current (SCLC) model [42]. The whole HRS process completes the obvious transition from ohmic conductivity to space charge-limited conductivity. This transition process conforms to the theoretical mechanism of space charge-limited current transfer due to the existence of traps [43,44].

Egg white is composed of 88.5% water, 10.5% protein, and 1% carbohydrates and inorganic ions. At present, 158 kinds of proteins can be identified, including ovalbumin, ovotransferrin, ovomucin, ovalbumin, globulin, histone, and two or more unique peptides [45]. Ovotransferrin is an iron-binding protein that is extremely sensitive to heat. The capture/release of charge causes the oxidation/reduction of iron ions in ferritin. Using the exchange characteristics of the ion itself, iron ions can be easily replaced by different kinds of transition metal ions [46]. When a negative voltage is applied, electrons are injected into the active layer through the upper electrode Al filling in the defects of charge trapping in the amino acids broken down by the protein and acting as a space charge. Since the work function difference between iron and aluminium or indium tin oxide electrodes is relatively small, oxidized iron ions can easily obtain electrons and be reduced to iron atoms. To explore whether the upper electrode of the device participates in the formation of conductive filaments, the electrons were introduced to the oxidized iron ions, which were easily reduced. We introduced Ag as the upper electrode to make Ag/EA: 1.2 ωt% MWCNT/ITO devices and tested the IV curve, as shown in Figure 6d. The electrical scanning characteristics of Ag/EA: 1.2 ωt% MWCNTs/ITO and Al/EA: 1.2 ωt% MWCNTs/ITO are consistent, which proves that the upper electrode does not participate in the formation of conductive filaments.

The resistance switching mechanism of the Al/EA:MWCNT/ITO devices can be schematically explained as follows. As shown in Figure 6e, when no bias is applied, the particles in the active layer are randomly distributed. When a negative bias is applied to the upper electrode Al, electrons enter the active layer from the upper electrode Al to fill the traps, and the oxygen ions in the active layer are affected by the electric field force and move to the bottom electrode, leaving oxygen vacancies. Some of the oxidized iron ions are affected by the electric field force and move to the upper electrode, where they are reduced by electrons (Figure 6f). As shown in Figure 6g, as the applied negative voltage increases, the electric field increases, generating an increasing number of oxygen vacancies and iron atoms. Finally, a conductive filament that connects oxygen vacancies and iron ions is formed between the upper and lower electrodes. At this time, the voltage reaches Vset, and the device changes from an HRS to an LRS. When a positive bias is applied to the upper electrode, the oxygen ions in the active layer are affected by the electric field force and move to the upper electrode Al to continuously fill the oxygen vacancies, and iron ions lose electrons and are further oxidized. As shown in Figure 6h, as the positive voltage increases, the oxygen vacancies and iron ions are continuously reduced with the enhancement of the electric field until the conductive filaments are destroyed. At this time, the voltage reaches Vreset, and the device changes from an LRS to an HRS.

MWCNTs have a strong ability to attract electrons, which can capture and transport electrons in the active layer. When the Al electrode is negatively biased, the electrons from the Al electrode and those in the active layer both fill the charge trapping centre and are trapped by the MWCNTs. The electrons under low bias voltage do not have enough energy/mobility to escape from the trapping centre [47]. The trapping centre is the electron trap centre, that is, the oxygen vacancy [48]. When the voltage reaches Vset, most of the charge trapping centres are filled with electrons, and the carriers increase rapidly. Percolation pathways of charge carriers are formed between the upper and lower electrodes, allowing electrons to jump between the MWCNTs and be transported along them, and the device is transformed into an LRS. At this time, electrons can move freely between the two electrodes, and the device forms a conductive filament [49]. After the reverse voltage is applied, the electrons are removed from the trap, and the device changes from an LRS to an HRS.

The content of MWCNTs in the composite film and the distance between them determine the trapping of carriers and the migration between MWCNTs. Therefore, as the concentration of MWCNTs incorporated in the egg white membrane increases, the distance between the MWCNTs decreases. The distance that electrons jump between MWCNTs decreases, the number of electrons in the composite film increases, and the conductivity of the RRAM increases. Due to the excessive content of MWCNTs, the Al/EA: 6 ωt% MWCNT/ITO devices did not exhibit resistance switching behaviour because they were densely packed in the composite film. This film could effectively transport charge carriers even under low bias, so that the device was always highly conductive.

## 4. Conclusions

In this paper, an Al/EA: MWCNT/ITO structured memristor was demonstrated. The current on-off ratio and reset voltage of the device could be adjusted by changing the concentration of MWCNTs in the active composite film. Al/EA/ITO devices and Al/EA:(1.2 ωt%, 3 ωt%, and 6 ωt%) MWCNT/ITO devices were fabricated. Analysis of their I-V characteristics proved that they could complete more than 100 consecutive bipolar resistance conversions in the same cell. The holding time reached 10^4^ s under a voltage of 0.1 V, and the switching window remained clear under 2.5 × 10^4^ continuous pulses. Statistical analysis of the Vset, Vreset, R_HRS_, and R_LRS_ of the device shows the improved reliability of the memristor formed by egg albumen doped with MWCNTs. The device can obtain different R_LRS_ states by adjusting the limiting current, and the multilevel resistance realizes 2-bit data storage to increase the storage density. The data can be continuously rewritten/erased 30 times and kept for 10^4^ s. The charge transfer mechanism of the device was based on the conductive principle of oxygen vacancy conductive filaments as well as iron atom conductive filaments. The increase in the concentration of carbon nanotubes resulted in an increase in the oxygen-containing functional groups, which promoted the formation of conductive channels to adjust the switching ratio of the device. In summary, Al/EA: MWCNT/ITO devices have excellent electrical characteristics, durability, stability, and reliability, allowing for potential applications in nonvolatile rewritable flash memory.
